# Sanitary Control Work

**Published:** 1901-11

**Authors:** 


### COMMUNICATIONS.

### SANITARY CONTROL WORK.

### Regulations Concerning Cattle Transportation.

*Restrictions Modified.*

United States Department of Agriculture,
Washington, D. C., October 22, 1901.

It is hereby ordered that section 3 of Bureau of Animal Industry Order No. 80, dated December 10, 1900, providing for the movement of cattle from the quarantined districts described by said order and amendments thereto, be amended as follows :

From November 15, 1901, to January 31, 1902, inclusive, cattle from said area may be moved to such points within the States of Virginia, North Carolina, Tennessee, Missouri, Kansas, and Texas, and the Territories of Oklahoma, New Mexico, and Arizona, as may be provided for in the regulations of these States and Territories, and permitted by the local authorities in charge. In the absence of such local regulations and permission, all movements of cattle from the quarantined districts to points outside of said district in the above-named States and Territories is prohibited. All cattle from the quarantined district destined to points outside of the States and Territories above named may be shipped without inspection between November 15, 1901, and January 31, 1902, inclusive, and without restrictions other than may be enforced by local regulations at point of destination. The reshipment of any cattle which may have been moved under this order to any part or parts of the States of Virginia, North Carolina, Tennessee, Missouri, Kansas, and Texas, and the Territories of Oklahoma, New Mexico, and Arizona, except by permission of the proper authorities of said States and Territories, is hereby prohibited.

And it is further ordered that all stock-pens which may have been reserved for the use of cattle from the quarantined district, prior to November 15th next, shall not be used for receiving or storing cattle from the quarantined district which have been inspected and passed, nor for cattle originating outside of the quarantined district, except when such cattle are intended for immediate slaughter.

